# Breast Tumor Detection and Diagnosis Using an Improved Faster R-CNN in DCE-MRI

**DOI:** 10.3390/bioengineering11121217

**Published:** 2024-12-01

**Authors:** Haitian Gui, Han Jiao, Li Li, Xinhua Jiang, Tao Su, Zhiyong Pang

**Affiliations:** 1 School of Biomedical Engineering, Shenzhen Campus of Sun Yat-sen University, Shenzhen 518107, China; guiht@mail2.sysu.edu.cn; 2 School of Electronics and Information Technology, Sun Yat-sen University, Guangzhou 510006, China; aqjiaoh@163.com; 3 State Key Laboratory of Oncology in South China, Collaborative Innovation Center for Cancer Medicine, Department of Medical Imaging, Sun Yat-sen University Cancer Center (SYSUCC), Guangzhou 510060, China; lil@sysucc.org.cn (L.L.); jiangxh@sysucc.org.cn (X.J.)

**Keywords:** breast cancer detection, deep learning, AI assistant

## Abstract

AI-based breast cancer detection can improve the sensitivity and specificity of detection, especially for small lesions, which has clinical value in realizing early detection and treatment so as to reduce mortality. The two-stage detection network performs well; however, it adopts an imprecise ROI during classification, which can easily include surrounding tumor tissues. Additionally, fuzzy noise is a significant contributor to false positives. We adopted Faster RCNN as the architecture, introduced ROI aligning to minimize quantization errors and feature pyramid network (FPN) to extract different resolution features, added a bounding box quadratic regression feature map extraction network and three convolutional layers to reduce interference from tumor surrounding information, and extracted more accurate and deeper feature maps. Our approach outperformed Faster R-CNN, Mask R-CNN, and YOLOv9 in breast cancer detection across 485 internal cases. We achieved superior performance in mAP, sensitivity, and false positive rate ((0.752, 0.950, 0.133) vs. (0.711, 0.950, 0.200) vs. (0.718, 0.880, 0.120) vs. (0.658, 0.680, 405)), which represents a 38.5% reduction in false positives compared to manual detection. Additionally, in a public dataset of 220 cases, our model also demonstrated the best performance. It showed improved sensitivity and specificity, effectively assisting doctors in diagnosing cancer.

## 1. Introduction

Breast cancer is the most frequently diagnosed cancer among women [1]. It is increasingly affecting younger individuals. Between 1985–1999 and 2010–2014, there has been a more significant rise in breast cancer prevalence among subjects aged 15–40 (16.3% to 33.9%) compared to those over 40 (43.6% to 57.9%) [2]. Fortunately, early detection has proven effective in reducing breast cancer mortality rates. And the large-scale breast screening programs are a crucial strategy for breast cancer early detection.

Breast magnetic resonance imaging (MRI) has proven valuable in breast cancer screening for high-risk populations with dense breasts [3]. It aids in differentiating between benign and malignant tumors, preoperative evaluation of breast cancer patients, and diagnosing occult breast cancer with axillary lymph node metastasis. Additionally, breast MRI can detect small and early-stage breast cancers that may be undetected by mammography, ultrasound, and physical examination. Dynamic enhanced MRI (DCE-MRI) of the breast can potentially assess microvessel density and contrast agent penetration rate, providing direct insights into the blood supply of breast tumors. This technique evaluates the morphological and hemodynamic characteristics of the entire breast tissue, facilitating the diagnosis of benign and malignant breast tumors.

In breast cancer screening, deep learning approaches were popular in breast segmentation, ROI detection, tumor segmentation and classification in MRI [4,5,6,7,8,9,10,11], ultrasound images [12,13,14], histopathological images [15,16], Digital Breast Tomosynthesis (DBT) [17] and DM [18,19,20,21,22,23,24]. Many limitations in breast MRI tumor detection and diagnosis have also been found in related studies (Table 1). Weakly supervised approaches [14,25], which classified the whole MRI scan first and then located the tumor through the heat map, were low accuracy because of the rough ROI. Strongly supervised object detection approaches such as Faster R-CNN [26] and Mask R-CNN [27] could perform object detection very well. These networks focus on the construction, shape, and texture while ignoring the glitches and information around the tumor that are essential for breast lesion diagnosis through deep learning approaches [28]. Though they performed well in breast tumor detection, they performed poorly in tumor diagnosis. In addition, dataset imbalance, background noise, and interference of other organs are challenges for deep learning approaches in automatic tumor detection and diagnosis [29].

Breast cancer can be manifested as irregular mass enhancement, star-shaped, linear, or branch-shaped enhancement. At the same time, benign lesions are mostly round or lobulated enhancements with clear boundaries, and the enhancement is more uniform. However, due to the high sensitivity of MRI, dense breast tissue, fat breast tissue, and background parenchymal enhancement (BPE) may mask tumors, leading to abnormal signals on imaging. Specific benign lesions (such as fibroadenomas or breast hyperplasia) may exhibit similar characteristics to malignant tumors on MRI, increasing the possibility of false positives. When an imprecise ROI contains benign tumors and some interference information from other tissues, it will seriously impact the classification performance of deep learning networks, reduce accuracy, and result in high false positives.

In this study, to obtain a more precise ROI and reduce the interference of the other tissues, a novel, precise deep network (PDN) for extracting the precise and deeper features was proposed and combined with the advantages of the Faster R-CNN and Mask R-CNN to implement the breast tumor detection and diagnosis. We denoised the MRI scans and segmented the breast with the U-net++ first [30]. We adopted the Vgg16, region proposal network (RPN) [26], feature pyramid network (FPN) [31], and ROI aligning for breast tumor detection. Lastly, we implemented the PDN to make the final diagnosis.

## 2. Materials and Methods

### 2.1. Datasets

The dataset included the international public breast dataset from Duke [32] and our internal private breast dataset from Sun Yat-sen University Cancer Center (SYSUCC). The breast MRI scans in the internal dataset were obtained using different MR scan equipment, such as GE medical systems SIGNA EXCITE and GE medical systems SIGNA HDx, at either 1.5T or 3.0T field strengths. Each DCE-MRI scan consisted of one unenhanced sequence and eight to 14 contrast-enhanced sequences.

In our breast segmentation work, we used a combination of the Duke dataset and our internal dataset to effectively train the U-net++ model. For breast cancer detection, our internal dataset consisted of 487 lesions, with 219 being malignant and 266 being benign cases. We used 277 lesions for training, 90 for validation, and 120 for testing. The malignant lesions included various types such as invasive cancer grades I, II, and III, ductal carcinoma in situ, semi-invasive carcinoma in situ, and nine other types of lesions. The benign lesions included fibroadenoma, fibrocystic breast disease, intraductal papilloma, and 19 different types of lesions. These lesions represented the common types of breast tumors. All lesions were confirmed through pathological biopsy or surgical pathology. The patients included in the study were consecutive cases collected between January 2007 and December 2020 at the cooperative hospital of Sun Yat-sen University Cancer Center in Guangzhou, China. The enrolled patients had a mean age of 49.1 years, ranging from 18 to 78 years. A nine-year experienced doctor verified the lesions. To validate our proposed method using an international public dataset, we used a hybrid dataset that included malignant cases from the Duke dataset and benign cases from our internal dataset. This approach was necessary since the Duke dataset only contained malignant cases, requiring us to combine it with our benign cases to evaluate the models. The Duke dataset comprised 100 malignant cases for training, 30 for validation, and 90 for testing. The dataset split and lesion information details are shown separately in Table 2 and Table 3.

### 2.2. Improved Faster R-CNN Architecture

**Figure 1:**
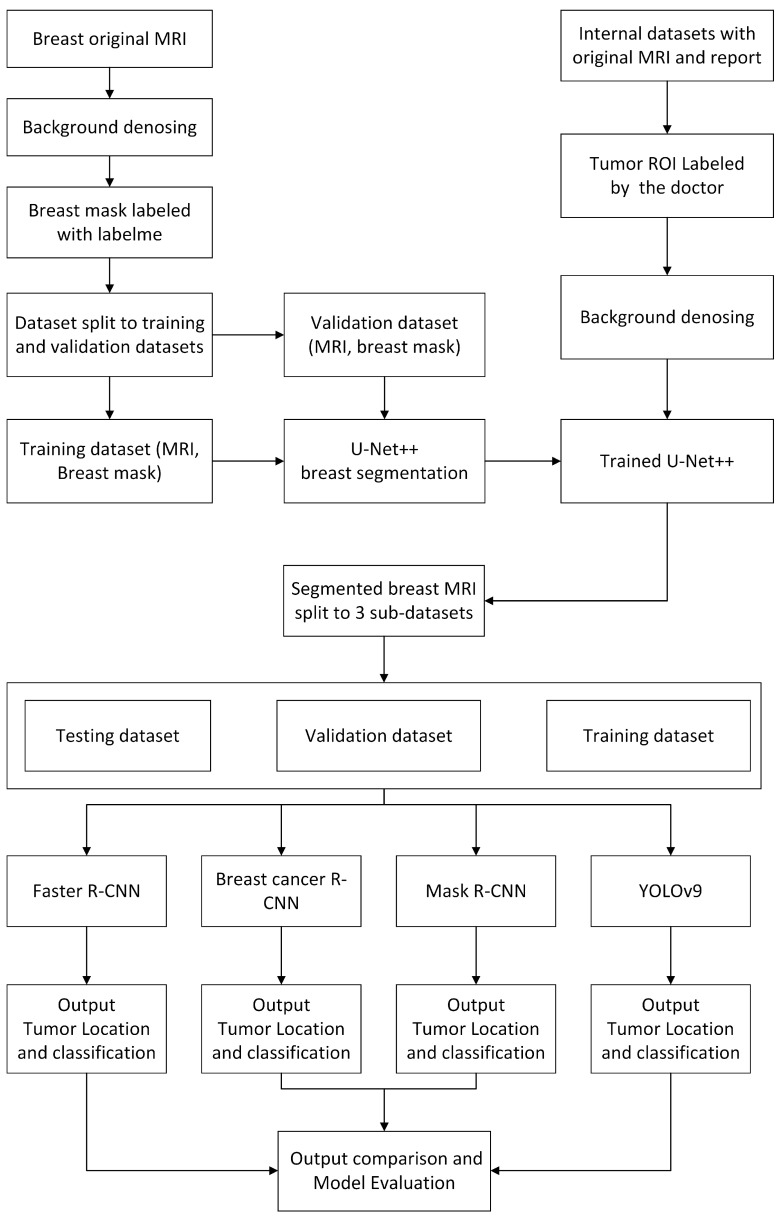
Flowchart of the study procedure.

As shown in Figure 1, Our study included breast segmentation and breast cancer detection. We used U-Net++ for breast segmentation, and the dataset consists of axial and sagittal breast MRIs and some single breast MRIs. It also included internal and external datasets, with 5–10 slices selected from different locations for each breast, some of which contained tumors, as well as dense and fat breast types. Labelme was used for polygon annotation and mask generation. The dataset was divided into training and testing sub-datasets after background denoising, and the Unet++ network was trained and tested. Regarding breast tumor detection and classification, the internal dataset was annotated by doctors using ITK-SNAP based on MRI reports. The Duke breast cancer cases have already been annotated. The trained Unet++ was used to segment the MRI after denoising. The segmented MRI and labeled coordinates were made into the VOC2007 dataset format. Finally, our model was trained and tested separately with Faster RCNN, Mask RCNN, and YOLOv9 [33] for comparative evaluation.

**Figure 2:**
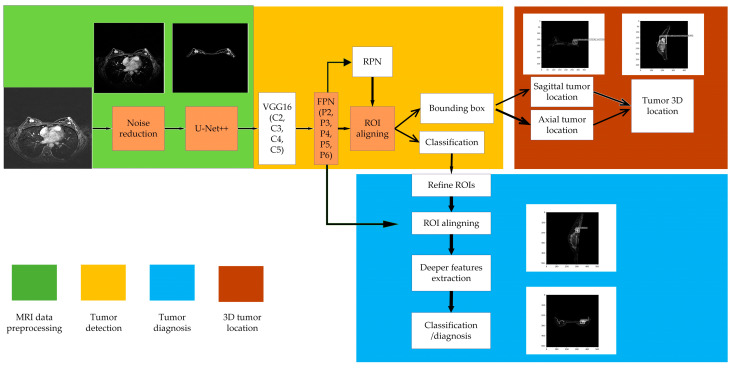
The architecture of our proposed model BC R-CNN.

The improved Faster R-CNN was customized for breast cancer detection, named breast cancer (BC) R-CNN, which included data preprocessing, tumor detection, and tumor diagnosis. The specific common features and differences with the original Faster R-CNN are shown in Table 4. Our system also supported tumor 3D location and tumor 3D diagnosis, as shown in Figure 2. The data preprocessing included background noise reduction and breast segmentation. Tumor detection had a VGG backbone, RPN, FPN, ROI aligning, and bounding box regression. FPN was optional in our study. Tumor diagnosis included ROI refining, deep feature extracting, and classification. Since we trained our model with sagittal and axial plane MRI, our system supported the 3D location and lesion-level diagnosis.

To eliminate the impact of redundant information and external noise, we denoised the MRI scan and used the well-trained U-Net++ model to preprocess images first. After that, the training and validation dataset only contained information about the breast region. We set the Intersection over Union (IOU) threshold to 0.5, the batch size to 1, the weight decay to 0.0005, and the initial learning rate to 0.00001. In the training subset, we trained 30 epochs with SGD optimization and a momentum of 0.9. Before training, the weights of pre-trained VGG16 of ImageNet were loaded for transfer learning, accelerating convergence.

We compared the test results with the doctor’s manual detection and the test results of the Faster R-CNN, Mask R-CNN, and YOLOv9 to evaluate the performance of our model.

The software environment was Python 3.8 with the open-source PyTorch library on a GPU-optimized workstation with a single NVIDIA GeForce RTX 3080Ti. After training the network, the best model was loaded to test all slices in the testing dataset. Each slice was standalone data. The model outputs two scores for each slice, representing the possibility of benign and malignant, respectively.

### 2.3. Image Preprocessing

The noise-reduced MRI was helpful for breast segmentation. To reduce the background noise in the MRI, all the pixels of the MRI were subtracted by a background value, which was the average value of the left-up block (4 × 4) pixels.

(1) $$Pixel_{new}=Pixel_{ori}-Pixel_{\mathrm{noise}}$$

(2) $${Pixel}_{\mathrm{noise}}=\frac{\sum_{i=0}^{N}\sum_{j=0}^{M}Pixel_{\mathrm{ij}}}{N\;\times \;M}$$

where the N and M were 4. was the coordinate (i, j) pixel value.

In breast MRI, the axial plane MRI was the standard position, containing two breasts. The sagittal plane MRI was beneficial for displaying Cooper’s ligament, the course of the mammary duct, and the axillary lymph nodes. Therefore, a combination of the sagittal and axial planes complemented each other and accurately located lesions. Since we had segmented the axial plane breast using U-Net++ [14] in a previous paper [10], we only expanded it to segment the sagittal plane breast and a single breast in this study. All the segmented breast images were scaled using a ratio of 512 divided by the shorter dimension (either the height or the width).

### 2.4. Tumor Detection

The tumor detection network was part of the Mask R-CNN; we retained the RPN, VGG16 backbone network, ROI aligning, the fully connected layers, and the bounding box regression from the Mask R-CNN. In addition, we employed the FPN as an optional feature and modified some parameters. Compared to deep learning networks that utilize single-scale feature maps, the FPN generated multi-scale feature maps by up-sampling them from deeper layers and combining them with the feature maps from the current layer. In the original paper, the FPN combined with the RPN generates 15 anchors at each level {P2, P3, P4, P5, P6} by assigning a single scale and width-to-height ratios of [1:2, 1:1, 2:1]. However, the limitation was that the feature maps differed for the fully connected layers in each scale layer. To address this, we assigned two neighbor scales to each level to obtain the optimal anchors. Furthermore, when generating the ROI feature map level, we reduced the standard ROI size from 224 to 160. All other parameters were set as described in the original paper.

### 2.5. Tumor Diagnosis

**Figure 3:**
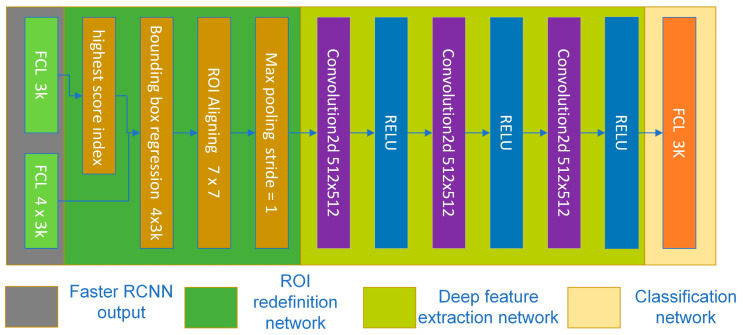
PDN structure.

Tumor diagnosis was based on the PDN, which incorporated three critical steps: refining the ROI, extracting deep feature maps, and performing classification (Figure 3). The original Faster R-CNN architecture consisted of two fully connected layers (FCLs). The first FCL generated 4 × K regression parameters, including two scales and two shift parameters, for each candidate, resulting in K ROI candidates. Our study focused on three ROI classes: benign tumor, malignant tumor, and background. We set the number of ROI candidates to 300, leading to a total of [300, 12] parameters for ROI regression. The second FCL was responsible for classifying all 300 ROI candidates. Each candidate had three scores representing the probability of each class. Therefore, there were [300, 3] classified scores in total. In the original Faster R-CNN architecture, the ROI after regression was the final ROI, and the highest classified score determined the last label.

To obtain accurate features in our study, we utilized the results from the second FCL as intermediate results for bounding box regression, enabling us to refine the ROIs with greater precision. In the PDN, we first extracted the class index with the highest score for each candidate and then performed ROI regression using the corresponding shift and scale parameters based on the class index. Secondly, we extracted the ROIs’ feature maps from the first stage’s complete feature maps [P2, P3, P4]. These corresponded to ROI sizes categorized as less than 64 pixels, between 64 and 128 pixels, and greater than 128 pixels. These feature maps were subsequently pooled to a fixed length of 7 × 7 using ROI Aligning and fed into deeper feature extractor layers. Finally, the deeper feature maps were fed into the last FCL to classify the 300 candidate ROIs into the background, benign, and malignant categories. The ROI label was determined by the class with the highest score. In our study, if the score for an ROI labeled as malignant exceeded the threshold of 0.5, it was considered a positive detection and retained for further analysis.

In addition, our proposed system supported breast tumor diagnosis by adopting information from both the sagittal plane MRI and the axial plane MRI. The final diagnosis of the lesion would be considered malignant if either of these MRI planes was classified as malignant.

### 2.6. Total Loss

Compared to the Faster R-CNN, the total loss function included an extra pdn_cls_loss, as shown in Equation (3). The pdn_cls_loss was the final classification cross-entropy loss in the PDN. It determined the discrepancy between the predicted class scores and the ground truth labels for the 300 candidate refined ROIs. It aided in accurately classifying the ROIs into the background, benign, or malignant tumor. Equation (4) was the loss of RPN, and Equation (5) was the loss of the second bounding box regressing layers. The rpn_cls_loss was the cross-entropy loss for classifying the foreground and background regions in the RPN. The rpn_loc_loss measured the discrepancy between the RPN’s predicted bounding box coordinates and the ground truth bounding box coordinates. The roi_loc_loss was the cross-entropy loss for the second bounding box regression. It calculated the difference between the predicted and refined bounding boxes obtained from the intermediate results. It further refines the ROIs. The roi_cls_loss was associated with classifying the ROI candidates obtained from the second FCL.

By incorporating the pdn_cls_loss into the overall loss function, the training process could optimize the network parameters to improve the accuracy and performance of the PDN in diagnosing breast tumors.

Loss_total = rpn_loss + roi_loss + pdn_cls_loss(3)

rpn_loss = rpn_loc_loss + rpn_cls_loss(4)

roi_loss = roi_loc_loss + roi_cls_loss(5)

### 2.7. Tumor 3D Location

**Figure 4:**
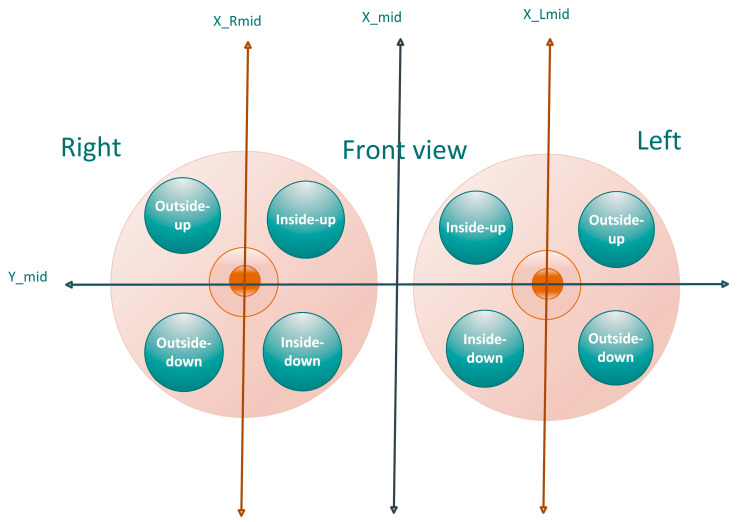
Four-quadrant location.

We located the tumor through the four-quadrant approach (Figure 4). The 3D center coordinates of the tumor were determined by calculating the coordinates of the ROI in the sagittal plane and axial plane separately. The process involved the following calculation steps:

(a). Axial baseline: The baseline of the breast in the axial plane.
(b). Middle sagittal line: The middle line of the two breasts in the sagittal plane.
(c). Middle lines of the right and left breast in the axial plane.
(d). Distance in the sagittal plane: The distance between the tumor and the center line of the breast in the sagittal plane.
(e). Distance to the baseline and middle line in the axial plane: The distance between the tumor and the baseline and the distance to the central line was calculated. These distances helped determine the quadrant of the breast where the cancer was located.

By following these steps, the 3D center coordinates of the tumor could be accurately determined, taking into account the breast’s anatomy and positioning in both the sagittal and axial planes.

(6) $$outside\_up=1{\;y}_{\mathrm{sag}}>Y_{mid}{\;\mathrm{and}\;x}_{\mathrm{ax}}<x_{Rmid}\;\mathrm{or}\;x_{ax}>x_{Lmid}$$

(7) $$outside\_down=1{\;y}_{\mathrm{sag}}<Y_{mid}{\;\mathrm{and}\;x}_{\mathrm{ax}}<x_{Rmid}\;\mathrm{or}\;x_{ax}>x_{Lmid}$$

(8) $$\mathrm{inside}\_up=1{\;y}_{\mathrm{sag}}>Y_{mid}\;\mathrm{and}\;x_{Rmid}<x_{ax}<x_{Lmid}$$

(9) $$\mathrm{inside}\_down=1{\;y}_{\mathrm{sag}}<Y_{mid}\;\mathrm{and}\;x_{Rmid}<x_{ax}<x_{Lmid}$$

(10) $$\left(x_{sag},y_{sag})=(\frac{x\_max_{\mathrm{sag}}+x\_\min_{sag}}{2},\frac{y\_\max_{sag}+y\_\min_{sag}}{2}\right)$$

(11) $$\left(x_{ax},y_{ax})=(\frac{x\_max_{\mathrm{ax}}+x\_\min_{ax}}{2},\frac{y\_\max_{ax}+y\_\min_{ax}}{2}\right)$$

where $\left(x_{sag},y_{sag}\right)$ is the center coordinate of the sagittal plane ROI and the $\left(x_{ax},y_{ax}\right)$ is the center coordinate of the axial plane ROI. The location is the left breast when $x_{ax}$ is less than $x_{\mathrm{mid}}$, which is the middle of the two breasts, and it is in the right breast. The location helps the doctor to write the report directly.

## 3. Results

### 3.1. Metrics

This study adopted the mean average precision (mAP50), true positive rate (TPR), false positive rate (FPR), recall (REC), sensitive (SENS), specificity (SPEC), Receiver Operating Characteristic Curve (ROC), and the area under the curve of ROC (AUC) as the metrics.

*TP* was the true positive, *TN* was the true negative, *FP* was the false positive, and *FN* was the false negative. The metrics were defined as follows:

(12) $$\mathrm{Recall}=\;\mathrm{Sensitive}=\mathrm{TPR}\;=\frac{TP}{TP+FN}$$

(13) $$Specificity=\frac{TF}{TF+FP}$$

(14) $$Precision=\frac{TP}{TP+FP}$$

(15) $$FPR=\frac{FP}{FP+TN}$$

ROC is a graph plotted with *TPR* as the *y*-axis and *FPR* as the *x*-axis, revealing the relationship between sensitivity and specificity (or specificity). The ROC curve calculates a series of sensitivity and specificity by setting multiple critical values for continuous variables, then plots the curve with sensitivity as the *y*-axis and (1-specificity) as the *x*-axis. The ROC curve has a wide range of applications in deep learning and machine learning, including performance evaluation, threshold selection, and model comparison. The area under the curve (AUC) under the ROC curve is an indicator for evaluating the performance of a model. The range of AUC values is between 0 and 1, with higher values indicating better model performance. AUC = 1 indicates a perfect classifier, while AUC < 0.5 indicates that the model performance is worse than random guessing. In addition, the optimal classification threshold can be selected using the ROC curve to achieve the best classification performance. In practical applications, different values may be necessary based on different needs, such as pursuing high sensitivity or high specificity. Lastly, when comparing different classification models, the ROC curve of each model can be plotted, and the model’s superiority can be evaluated by comparing the area under the curve (AUC).

MAP50 is a critical metric in object detection, which measures the average precision of the model at a lOU (Intersection over Union) threshold of 0.5. IOU is an indicator measuring the degree of overlap between predicted and real bounding boxes. It is usually calculated across multiple categories and averaged to obtain the overall average precision. For each category, the precision–recall curve is first calculated at a loU threshold of 0.5, then the area under the curve (AUC) is calculated, and finally, the average AUC for all categories is obtained.

### 3.2. Image Preprocessing Performance

**Figure 5:**
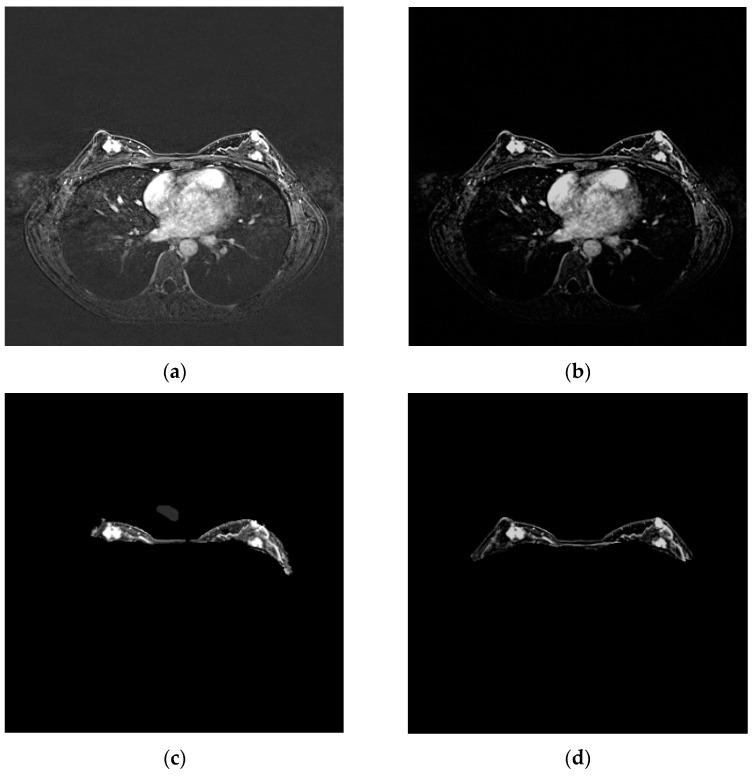
Background noise reduction: (**a**) MRI before noise reduction; (**b**) MRI after noise reduction; (**c**) segmented breast of original MRI; (**d**) segmented breast of noise reduction MRI.

**Figure 6:**
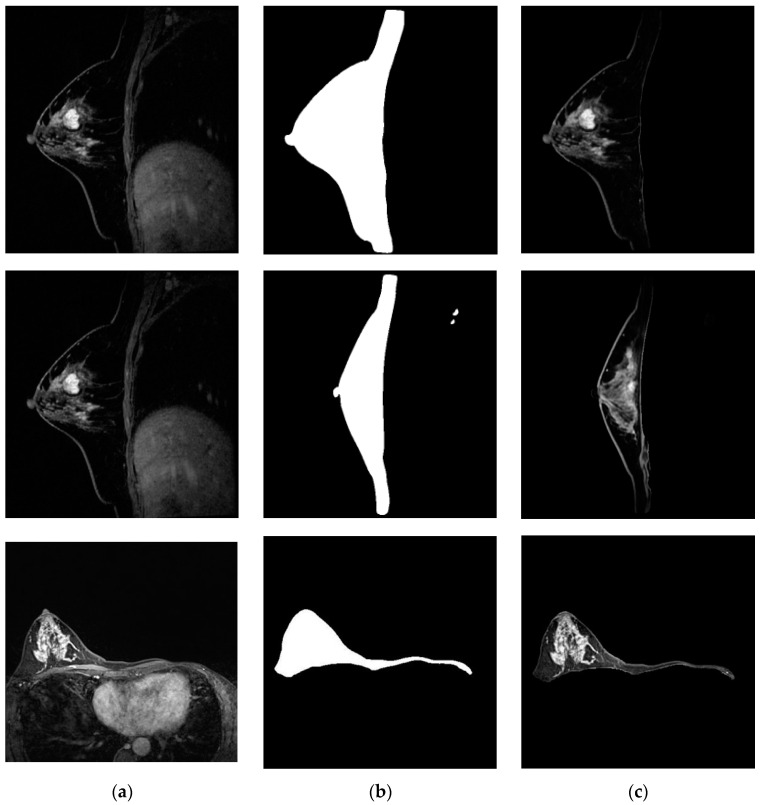
U-Net++ breast edge segmented: (**a**) sagittal breast MRI and single breast MRI at axial plane; (**b**) masks; (**c**) segmented breasts.

The segmented breast was more accurate and explicit after implementing the noise reduction to the original MRI scan (Figure 5). After the training, the U-Net++ could accurately segment the sagittal plane and single breast (Figure 6).

### 3.3. Breast Cancer Detection

**Figure 7:**
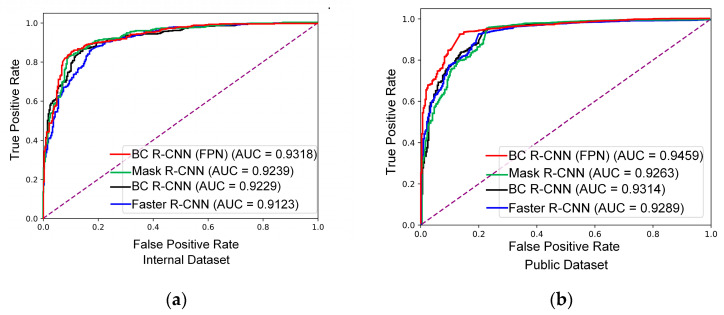
AUC performance comparison of different models: (**a**) internal dataset, (**b**) public dataset.

We evaluated the performance of breast cancer detection using lesion-level sensitivity and false positive, slice-level PREC, REC, MAP, and area under the receiver operating characteristic (ROC) curve (AUC) as the criteria. We adopted the highest score ROI and label from all the candidates. As shown in Table 5 and Figure 7, for the internal dataset, when the FPN was turned off, our model achieved a sensitivity of 0.9500 and a false positive rate of 0.1333; the false positive rate improved by 38.5% and 33.3% compared to doctor’s manual detection and Faster R-CNN, respectively. Compared to Mask R-CNN, the sensitivity was improved by 8.0%. For the Duke dataset, our mode achieved the highest sensitivity of 0.9743 and the lowest false positive rate of 0.1167 compared to the Faster R-CNN and the Mask R-CNN. The FPN did not improve the system’s performance for our dataset in this study. The network adding the PDN had significant improvement. In particular, the false positive rate improved a lot. The improved Faster R-CNN demonstrated its effectiveness in reducing the false positive rate of tumor detection in MRI images with noisy backgrounds.

**Figure 8:**
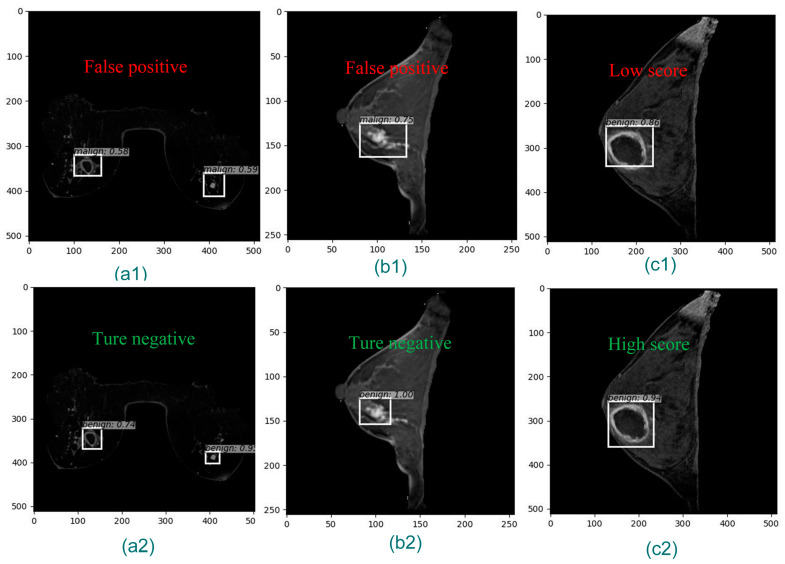
Breast tumor location and diagnosis comparison of Faster R-CNN and our proposed model: (**a1**,**b1**,**c1**) detected by Faster R-CNN; (**a2**,**b2**,**c2**) detected by our proposed model. (**a1**,**b1**) false positive; (**c1**) diagnosed with a lower score; (**c2**) diagnosed at a higher score.

Our model outperformed Faster R-CNN and Mask R-CNN with a more precise ROI and diagnosed at a lower positive rate (Figure 8).

## 4. Discussion

In this study, we successfully developed a novel and fully automatic breast cancer detection system using a single MRI scan based on deep learning techniques. Our system demonstrated superior accuracy and reliability in detecting and classifying breast tumors compared to the doctor’s manual breast cancer detection. This is particularly true given that all the benign tumors in this study were confirmed through pathological testing, with most of them presenting suspected malignant lesions. As a result, achieving an effective and accurate diagnosis of these lesions has become extremely difficult. Utilizing deep learning techniques and integrating various networks in our system have proven highly effective in enhancing accuracy and reducing the false positive rate of breast cancer diagnosis.

Our approach was strongly supervised deep learning. In comparison to weakly supervised methods [14,25], which considered the entire MRI scan as the ROI, extracted feature maps, and subsequently located the tumor using a heat map after classification, our system took a different approach that defined the ROI by employing a bounding box. This precise localization allowed us to extract highly accurate feature maps exclusively from the ROI. By focusing on the tumor region directly, we mitigated the issues associated with imprecise feature maps, which could negatively impact the classification performance. Additionally, the low accuracy of the heat map, generated in weakly supervised methods, could adversely affect the accuracy of tumor localization. By adopting a strongly supervised approach with precise ROI localization and subsequent feature map extraction, our system achieved higher accuracy, sensitivity, and specificity and reduced false positives.

The Faster R-CNN and Mask R-CNN models, as two-stage R-CNNs, had demonstrated strong performance in object detection and classification tasks. However, these models also had certain limitations. Firstly, these models extracted the features from an initial draft ROI but not from the final precise ROI, which led to decreased performance when the surrounding information of the object, such as breast cancer, was crucial for accurate classification. To address this issue, we proposed a PDN that utilized the initial classification result as intermediate data to select the corresponding class bounding box for the candidates to help the network focus on the glitches of the tumor. The feature maps fed to the classification layer were extracted from the precise ROI. The resulting feature maps were much more accurate and detailed than those obtained from the initial draft ROI. Our test results demonstrate improved performance, particularly in reducing the false positive ratio. Secondly, in Faster R-CNN and Mask R-CNN, the feature maps used for classification are the same as those used for object localization, designed for the entire MRI scan. This design choice prioritizes object location over object classification, which can affect the performance of tumor classification. To achieve better performance in tumor classification, we introduced three additional convolutional layers to extract deep features specifically from the ROIs, which helped our system focus on the discriminative features relevant to tumor classification, enhancing the overall performance of the cancer detection system.

YOLO’s models are excellent for real-time object detection. For example, the latest model, YOLOv9, is known for its high speed. One reason for this speed is that YOLO uses significantly fewer anchors compared to Faster R-CNN. YOLOv9 utilizes three anchors per feature point, whereas Faster R-CNN employs nine. This difference contributes to YOLO’s overall faster performance, making it ideal for real-time object detection applications with strict timing requirements. However, YOLOv9 also has some limitations, including lower accuracy and a higher false positive rate. Additionally, it has a greater rate of missed detection. Our experimental results from an internal dataset showed that YOLOv9 achieved a mean average precision (mAP) of 0.658, while our model achieved a mAP of 0.750. Breast MRI testing primarily focuses on diagnosing high-risk patients, detecting recessive breast cancer, evaluating and staging before surgery, monitoring postoperative outcomes, and tracking recurrence. These tasks do not have strict real-time requirements for breast cancer detection. Instead, they emphasize detection accuracy, aiming to minimize missed cases and reduce false positive rates, which is of greater clinical significance. From this perspective, our improved Faster R-CNN offers more practical value for breast cancer detection than YOLOv9.

Few studies have considered both the axial and sagittal MRI. The tumor sections observed in the sagittal plane can differ significantly from those in the axial plane. To leverage the information from both axial and sagittal plane MRI scans, we trained separate instances of the U-Net++ network to segment the breast in each plane. We then trained the network using a combination of MRI scans from both planes for tumor detection and classification. This approach allowed us to detect breast cancer in a single-plane MRI. We obtain more scientifically sound, reasonable, and reliable effects by considering the diagnostic results from both axial and sagittal planes, which provide different cross-sectional views. It was particularly robust for detecting lesions exhibiting significant characteristics in a single plane slice and identifying small lesions.

The accurate 3D location of breast tumors is crucial for doctors to diagnose and perform surgeries. However, most existing studies on tumor detection focused on 2D analysis in a single plane of the MRI. To improve the accuracy of tumor positioning, we proposed a method that derives the 3D location of the tumor by combining information from the axial and sagittal planes. This enhancement provided more precise tumor location information in the diagnostic report, aiding doctors in future inspections and surgical procedures.

Despite the improvements achieved, our study had several limitations. Firstly, we had to fine-tune the common convolutional layers using pre-trained parameters from ImageNet because of the limitation of the dataset, which help compensate for the small dataset but may introduce certain biases. Secondly, our study solely relied on the MRI spatial information and did not utilize the time signal intensity curve (TIC) and apparent diffusion coefficient (ADC). Incorporating these features could potentially enhance the accuracy of breast cancer detection. Lastly, we could have calculated the exact 3D volume of the lesions, leaving room for further improvement in terms of accuracy and efficiency. We plan to develop a multi-modal deep-learning breast cancer detection system incorporating ADC and TIC data in our future work. Additionally, we aim to expand our local dataset and incorporate more publicly available datasets further to enhance the performance and generalizability of our system. By addressing these limitations and considering additional features, we strive to provide a more robust and accurate breast cancer detection system that can assist medical professionals in making informed decisions and improving patient care.

## 5. Conclusions

Our study presented an improved Faster R-CNN, the first deep learning approach that focused on burr around the tumor in breast tumor detection and diagnosis. It improved sensitivity and reduced the false positive rate in breast cancer diagnosis by merging with a novel PDN and the advantages of existing two-stage object detection approaches. These advancements may potentially enhance breast cancer screening and ultimately improve patient outcomes in the diagnosis and treatment of breast cancer.

## Figures and Tables

**Table 1:** Summary of related studies on breast tumor detection and diagnosis.

References	Dataset	Deep Learning Approaches	ROI	Limitations
[5]	DCE-MRI: 339 malignant	Beast segmentation: no tumor; detection: Mask R-CNN tumor; diagnosis: Mask R-CNN; features: shared	Detection: whole image; diagnosis: bounding box	Low sensitive; high false positive rate: per-slice sensitive, false positive rate, accuracy, and AUC were 80%, 25%, 75%, and 0.71 separately; unprecise ROI; shared features.
[6]	DCE MRI: 75 malignant	Beast segmentation: U-Net++; detection: Faster R-CNN; diagnosis: no	Detection: segmented breast; diagnosis: no	Small dataset and only mass detection
[7]	DCE MRI: 52 benign, 76 malignant	Beast segmentation: no tumor; detection: no diagnosis: boosted random forest; features: radiomic and ADC	Detection: no; diagnosis: tumor	Sensitivity was 84.6% and positive predictive value was 78.6%; low sensitivity and high false positive rate.
[10]	DCE MRI: 45 benign, 27 malignant	Beast segmentation: no tumor; detection: no diagnosis: Resnet 50 + DC-LSTM; features: DCE and ADC	Detection: no; diagnosis: tumor	Low accuracy: accuracy was 0.847
[11]	DCE MRI: 161 malignant	Beast segmentation: U-net; tumor detection: U-net; diagnosis: 3D Dense CNN; features: separate	Detection: segmented breast; diagnosis: tumor	Low sensitivity (0.643)
[12]	Breast Ultrasound: 434 lesions	Beast segmentation: no tumor; detection: dense net + template mask; diagnosis: no	Detection: whole image; diagnosis: no	Mass detection only
[13]	Breast Ultrasound: 437 benign, 210 malignant	Beast segmentation: no; detection: LEDNet model; diagnosis: ResNet-18 + SEO RNN	Detection: whole image; diagnosis: tumor	High false positive rate
[18]	Digital breast tomosynthesis: 75 benign, 289 malignant	Beast segmentation: no tumor; detection: 3D Mask R-CNN; diagnosis: 3D Mask R-CNN; eatures: shared	Detection: whole image; diagnosis: bounding box	Imbalance dataset, only mass detection without diagnosis

**Table 2:** Dataset for breast segmentation and breast tumor detection and diagnosis.

Source	Task	Dataset	Training	Validation	Testing	Subtotal
SYSUCC	breast segmentation	Single breast	8		3	11
SYSUCC	breast segmentation	Sagittal plane	1180		235	1415
SYSUCC	breast segmentation	Axial plane	1070		383	1453
SYSUCC	breast segmentation	With visible tumor	1000		300	1300
SYSUCC	breast segmentation	Without visible tumor	1250		318	1568
DUKE	breast segmentation	With visible tumor	98		4850	4949
SYSUCC	tumor detection and diagnosis	Benign lesions	161	45	60	266
SYSUCC	tumor detection and diagnosis	Malignant lesions	114	45	60	219
SYSUCC	tumor detection and diagnosis	Benign slices	1269	259	329	1857
SYSUCC	tumor detection and diagnosis	Malignant slices	1043	341	453	1837
DUKE	tumor detection and diagnosis	Malignant lesions	100	30	90	220
DUKE	tumor detection and diagnosis	Malignant slices	2000	600	2000	4600

**Table 3:** Distribution of breast tumors in the dataset.

Source	Lesion Type	Lesion Name	Number
Duke	Malignant	Invasive cancer	230
SYSUCC	Malignant		
SYSUCC		Invasive cancer	142
SYSUCC		Ductal carcinoma in situ	53
SYSUCC		Semi-invasive carcinoma in situ	8
SYSUCC		Premium Executive Internal Cancer	5
SYSUCC		Invasive lobular carcinoma	5
SYSUCC		low-grade intraductal carcinoma	1
SYSUCC		Intermediate-grade intraductal carcinoma	1
SYSUCC		Intraductal papillary carcinoma	1
SYSUCC		Invasive intraductal carcinoma	1
SYSUCC		Squamous cell carcinoma	1
SYSUCC		Eczematous breast cancer	1
SYSUCC		Calcified intraductal carcinoma	1
SYSUCC	Benign		
SYSUCC		Fibroadenoma	160
SYSUCC		Fibrocystic breast disease	39
SYSUCC		Intraductal papilloma	33
SYSUCC		Adenopathy	7
SYSUCC		Papillary hyperplasia	4
SYSUCC		Hyperplastic nodule	3
SYSUCC		Lymphoma infiltration	3
SYSUCC		Lymphoma	3
SYSUCC		Chronic inflammation	2
SYSUCC		Fibroadenosis with lymphoid tissue	2
SYSUCC		Chronic granulomatous inflammation	1
SYSUCC		Complex sclerosing lesions	1
SYSUCC		Glandular hyperplasia with myoepithelial hyperplasia	1
SYSUCC		Interstitial fibrous collagen hyperplasia	1
SYSUCC		Myoepithelial tumor	1
SYSUCC		Calcified nodules	1
SYSUCC		Dermal fibroma	1
SYSUCC		Compliant with BI-RADS Class II	1
SYSUCC		Compliant with BI-RADS Class III	1
SYSUCC		Fibroadenoma with tubular adenoma	1
SYSUCC		Epithelial hyperplasia	1

**Table 4:** The common features and differences between our model and the original Faster R-CNN.

Items	Original Faster R-CNN	Improved Faster R-CNN	Reasons
RPN	yes	Yes	Region proposals
FPN	No	Yes	Multi-scale feature extractionImprove small object detection performance
PDN	no	yes	Narrow the ROIs and reduce the interference
Common convolutional layers	16	16	Extract common features
Extra convolutionoanl layers for classification	0	3	Extract the extra deeper features
Method of extract features from ROI	ROI Pooling	ROI aligning	Sample ROI features more accurately, reducing errors caused by quantization
Feature maps for location	ROI from first bounding box regression	ROI from first bounding box regression	The final location is the second regression bounding box
Feature maps for classification	ROI from first bounding box regression	ROI from second bounding box regression	more accurate features

**Table 5:** Performance of our proposed model BC R-CNN, Faster R-CNN, Mask R-CNN, and doctor’s manual approach in breast tumor detection and diagnosis for internal and international datasets.

Dataset	Network	FPN	PDN	Map	PREC (M)	REC (M)	PREC (B)	REC (B)	AUC	Sensitivity	False Positive
SYSUCC	Manual detection									0.9830	0.2167
SYSUCC	YOLOv9	No	No	0.6580	0.6390	0.6160	0.5500	0.7520	0.6620	0.6840	0.405
SYSUCC	Mask R-CNN	Yes	No	0.7175	0.8891	0.7897	0.7479	0.8440	0.9239	0.8800	0.1200
SYSUCC	BC R-CNN (FPN)	Yes	Yes	0.7302	0.9241	0.7897	0.7500	0.8715	0.9318	0.9333	0.1833
SYSUCC	Faster R-CNN	No	No	0.7113	0.8374	0.8299	0.7791	0.7553	0.9123	0.9500	0.2000
SYSUCC	BC R-CNN	No	Yes	0.7524	0.8826	0.8076	0.7507	0.8104	0.9299	0.9500	0.1333
Duke	YOLOv9	No	No	0.693	0.7150	0.6640	0.7100	0.6020	0.6380	0.6330	0.2880
Duke	Mask R-CNN	Yes	No	0.7125	0.8998	0.8469	0.6910	0.7204	0.9263	0.9658	0.2000
Duke	BC R-CNN (FPN)	Yes	Yes	0.7224	0.9283	0.8061	0.5786	0.8054	0.9459	0.9572	0.1833
Duke	Faster R-CNN	No	No	0.6726	0.9019	0.8357	0.5990	0.7445	0.9289	0.9572	0.1500
Duke	BC R-CNN	No	Yes	0.7057	0.9126	0.8474	0.6495	0.7325	0.9314	0.9743	0.1167

M, malignant tumors; B, benign tumors.

## Data Availability

DCE-MRI data used to support the findings of this study were supplied by the Sun Yat-sen University Cancer Center (Guangzhou, China) under license and have not been made freely available because of patient privacy. If our dataset is helpful to you, please contact the corresponding author by e-mail.
